# Upper Eyelid Static Surgical Approaches for the Treatment of Facial Palsy-Induced Lagophthalmos: A Systematic Review

**DOI:** 10.3390/jcm14134688

**Published:** 2025-07-02

**Authors:** Giovanni Ottonelli, Jacopo Celada Ballanti, Alessandro Gaeta, Gianmaria Barone, Novella Montericcio, Alessandra Di Maria

**Affiliations:** 1 Department of Ophthalmology, Humanitas University, Pieve Emanuele, 20090 Milan, Italy; giovanni.ottonelli@humanitas.it (G.O.); jacopo.celadaballanti@humanitas.it (J.C.B.); gianmaria.barone@humanitas.it (G.B.); 2 Department of Internal Medicine and Medical Specialties (DIMI), Università di Genova, Viale Benedetto XV, 16132 Genova, Italy; alessandrogaeta01@gmail.com; 3 Biomedicine, Neuroscience and Advance Diagnostic (BIND) Department, University of Palermo, 90133 Palermo, Italy; novella.montericcio@gmail.com; 4 Department of Ophthalmology, IRCCS Humanitas Research Hospital, Rozzano, 20089 Milan, Italy

**Keywords:** paralytic lagophthalmos, facial palsy, eyelid loading, levator palpebrae superioris, LPS, levator retraction, retraction, gold weight, platinum chain, autologous fat grafting, lipofilling, müllerectomy, static upper lid surgery

## Abstract

**Background**: Incomplete eyelid closure and lagophthalmos due to facial nerve palsy are significant functional and aesthetic concerns often requiring surgical correction. The aim of this systematic review is to quantitatively assess the efficacy, safety, and patient satisfaction associated with gold or platinum weight implantation, autologous fat grafting (lipofilling), and müllerectomy. **Methods**: A systematic review was performed following PRISMA guidelines, searching PubMed, Embase, Cochrane Library, Web of Science, and Scopus up to March 2025. Studies included clinical data on surgical correction for incomplete eyelid closure in facial palsy, reporting functional, anatomical, and satisfaction outcomes. Quality was assessed using the Newcastle–Ottawa Scale (NOS) and the Grading of Recommendations Assessment, Development, and Evaluation (GRADE) system. **Results**: Twenty-six studies including a total of 1205 patients were included. Gold/platinum weight implantation achieved complete or near-complete eyelid closure in 83–92% of cases, with a reduction in lagophthalmos to <1 mm. Complication rates ranged from 5–15% (mainly extrusion/migration), and patient satisfaction averaged 7.9/10. Lipofilling showed persistent benefit in 77% of cases, with 9–20% requiring repeat procedures and 10–12% experiencing minor complications. Müllerectomy yielded symptomatic improvement or resolution in 92% of cases, with a mean lagophthalmos reduction of 1.18 mm. **Conclusions**: Gold or platinum weight implantation provides the most reliable improvement for severe upper eyelid dysfunction in facial palsy. Lipofilling is a viable autologous alternative, while müllerectomy is effective in selected cases. Further prospective comparative trials are needed to refine surgical selection and optimize outcomes.

## 1. Introduction

Proper eyelid function is essential for maintaining ocular surface health, tear film stability, and visual clarity. Eyelid opening and closure rely on a finely tuned interaction between antagonistic muscle groups. Elevation of the upper eyelid is primarily mediated by the levator palpebrae superioris (LPS) muscle, innervated by the superior division of the oculomotor nerve, with secondary support from Müller’s muscle [1,2]. Eyelid closure, in contrast, is controlled by the orbicularis oculi muscle, a circumferential muscle innervated by the facial nerve (cranial nerve VII). Together, these muscles regulate both voluntary and involuntary blinking, which are critical for distributing the tear film, removing debris, and protecting the corneal epithelium from environmental insults [3].

Disruption of this coordination can result in incomplete eyelid closure and lagophthalmos. When the orbicularis oculi is paralyzed, as in facial nerve palsy, unopposed activity of the LPS leads to ineffective eyelid closure and persistent corneal exposure. This dysfunction may cause significant ocular surface morbidity, including dryness, irritation, exposure keratopathy, and in advanced cases, corneal ulceration or vision loss [4]. Additionally, the facial asymmetry associated with eyelid dysfunction can significantly impact psychosocial well-being and quality of life [5].

Surgical intervention is typically indicated in moderate to severe cases of lagophthalmos, particularly when conservative approaches fail or when patients experience corneal exposure or psychosocial distress related to appearance [6]. Various surgical techniques have been described for the treatment of lagophthalmos, including levator recession, tissue lengthening, and implant- or volume-based procedures [7,8]. Broadly speaking, the operative options can be divided into two mechanistic groups: static and dynamic. Static procedures work by adding weight, length, or volume to the upper lid so that gravity or passive tension ensure closure; on the contrary, dynamic procedures aim to restore active blink by re-establishing neuromuscular drive (e.g., palpebral springs, temporalis muscle transfer, cross-facial nerve grafts) [9,10]. Because static upper lid loading and volume-augmentation techniques constitute the current first-line surgical approach for facial palsy-induced lagophthalmos, the present review is confined to that category, while dynamic reconstructions are discussed only briefly in the context of excluded alternatives (see Section 4.1).

Among static procedures, autologous fat grafting (lipofilling) and upper eyelid weight loading are widely used and well-established approaches. Lipofilling involves harvesting, processing, and reinjecting the patient’s own adipose tissue into the upper eyelid. Beyond volume restoration, this technique may offer regenerative benefits via adipose-derived stem cells [11]. Its biocompatibility, low immunogenicity, and potential for natural contour restoration make it an appealing option for both functional and aesthetic correction [12,13].

Weight implantation, typically using gold or platinum inserts placed in the pretarsal space, enhances passive eyelid closure by harnessing gravitational force [14]. This technique provides immediate and predictable results, though it may be associated with complications such as implant migration, extrusion, foreign body sensation, or hypersensitivity reactions [15]. As such, surgical approaches that minimize tissue disruption and preserve periorbital anatomy are increasingly emphasized [16].

A less frequently used but potentially useful adjunct is müllerectomy, which involves the excision or recession of Müller’s muscle. In patients with coexisting upper eyelid retraction or mild lagophthalmos, particularly where Müller’s muscle is hyperactive or fibrotic, this approach can reduce eyelid height without altering levator function [17]. When combined with levator recession, müllerectomy may be beneficial in complex or asymmetric presentations [18].

Despite the widespread use of these techniques, there is a lack of direct, head-to-head comparative studies on lipofilling, gold weight implantation, and müllerectomy for lagophthalmos and incomplete eyelid closure correction. Given the increasing demand for both functional and aesthetic results in periocular surgery, a systematic review is warranted to evaluate their efficacy, safety profiles, patient satisfaction, and complication rates. This review, while exploring all current surgical possibilities of lagophthalmos correction in facial palsy, aims to consolidate the current evidence to support clinical decision-making and guide the optimization of surgical strategies for incomplete eyelid closure.

## 2. Materials and Methods

**Figure 1:**
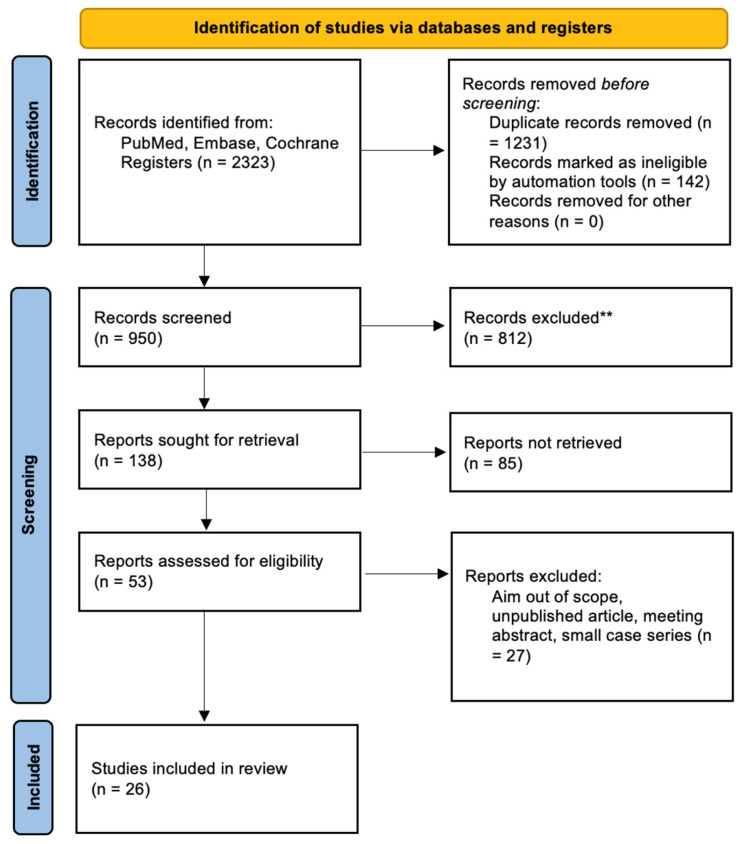
Prisma flowchart. ** All exclusions during the screening phase were carried out manually by human reviewers.

A systematic review was performed to determine the current surgical approaches for correcting incomplete eyelid closure in patients with facial nerve palsy. In conducting this review, we followed the Preferred Reporting Items for Systematic Reviews and Meta-Analyses (PRISMA) 2020 guidelines, which provide a structured framework to enhance the transparency and completeness of systematic reviews. We reported key methodological elements including the eligibility criteria, information sources, and the study selection and data extraction processes. The PRISMA flow diagram was used to depict the screening and inclusion process (see Figure 1).

To identify relevant articles, we conducted a systematic literature search in March 2025 using a controlled vocabulary and specific keywords. Specifically, we used the following research string “(facial paralysis) OR (facial palsy) OR (eyelid retraction) OR (facial nerve paralysis) OR (incomplete eyelid closure) OR (lagophthalmos) AND (surgical correction) OR (eyelid surgery)”. The search was conducted in electronic databases such as PubMed, Embase, the Cochrane Library, Web of Science, and Scopus. “Rayyan” software (version 2022, Cambridge, MA, USA, 2022) was used as an automation tool to assist in the selection process [19]. The complete search strategy is given in Appendix A.

After compiling the electronic list of articles and ensuring the automatic exclusion of duplicates, two reviewers (A.G. and J.C.B.) independently screened all the abstracts to identify studies that met the inclusion criteria. This process included all clinical studies that evaluated surgical interventions for correcting incomplete eyelid closure due to facial nerve palsy, with outcomes related to functional or anatomical improvement. The exclusion criteria were as follows: eyelid retraction unrelated to facial nerve palsy (e.g., thyroid eye disease), studies focusing exclusively on non-surgical treatments (e.g., botulinum toxin, fillers), lower eyelid or non-eyelid procedures, lack of relevant outcome data or case reports, case series with fewer than ten patients, animal or cadaveric studies, editorials, reviews, letters, systematic reviews, meta-analyses, and studies published in languages other than English without an available translation.

In refining our eligibility criteria, we limited the review to static upper lid methods that augment eyelid mass or volume. Dynamic palpebral springs were therefore excluded because they aim to restore active blink. Likewise posterior lamellar spacer grafts were omitted because they are used chiefly for thyroid-related upper lid retraction or lower lid ectropion rather than facial palsy lagophthalmos (see Section 4.1).

The specific inclusion and exclusion criteria are reported in Appendix B. After this first screen the same two authors (A.G. and J.C.B.) independently performed a full-text screening of the selected articles, obtaining a final group of feasible papers. At this point a consensus was reached in cases of disagreement among reviewers, and two expert reviewers (O.G. and G.B.) were consulted if necessary to provide additional expertise. The determining reasons for the inclusion or exclusion of the full-text reviewed articles are summarized in Appendix C. In the cases of disagreements during the selection process, the two reviewers resolved the conflicts through direct discussion. No unpublished data were requested from the authors of the included studies; the analysis was based solely on the available published information.

These recommendations provide a methodological framework to evaluate the reliability and validity of evidence in medical research. The risk of bias was independently evaluated by two distinct authors (A.G. and J.C.B.) for every included study with the Newcastle–Ottawa Scale [20]; two reviewers scored each domain and resolved any disagreements through consensus. Overall, the assessment found predominantly moderate risks of bias, chiefly stemming from retrospective designs and incomplete outcome reporting (see Table 1). The Grading of Recommendations Assessment, Development, and Evaluation (GRADE) system [21] was applied to evaluate the overall quality of the included studies and to facilitate the formulation of evidence-based conclusions.

Figure 1 summarizes the research approach applied in this systematic review within a PRISMA flowchart.

## 3. Results

This systematic review analyzed data from 26 studies [22,23,24,25,26,27,28,29,30,31,32,33,34,35,36,37,38,39,40,41,42,43,44,45,46,47] encompassing a total of 1275 male and female patients undergoing surgical management for upper eyelid retraction and lagophthalmos. The investigated techniques included upper eyelid weight implantation (in most cases) [22,23,24,25,26,27,28,29,30,31,32,33,34,35,36,37,38,39,40,41,42,43,44], autologous lipofilling (fat grafting) [45,46], and müllerectomy [47]. A subset of studies directly compared these surgical interventions, with particular attention to functional restoration, aesthetic outcome, complication profile, and patient satisfaction.

Patient ages ranged from 6 to 92 years, with the average age across the studies in the mid-50s, aligning with the epidemiology of facial nerve paralysis underlying upper eyelid retraction and lagophthalmos. In some studies, the number of eyes exceeded the number of patients, reflecting bilateral involvement. The study sample sizes varied widely, from as few as 10 to as many as 130 patients, with both male and female participants represented, and ages ranging from adolescence to late adulthood. This demographic overview sets the context for analyzing and comparing surgical treatments for incomplete eyelid closure, focusing on weight implantation, autologous fat grafting (lipofilling), and müllerectomy, each with distinct indications and outcome profiles. The underlying etiologies were diverse: idiopathic facial nerve palsy (Bell’s palsy) accounted for the majority (up to 72% in some cohorts [36]), followed by postsurgical, post-traumatic, and, less frequently, congenital cases. The follow-up periods ranged from a minimum of 2 months to 10 years, with mean/median follow-up typically between 12 and 48 months. Most studies provided at least 6 months of follow-up, with several reporting on durability beyond 5 years. Across all the studies, surgical candidates were those with symptomatic lagophthalmos (mean preop gap 4.5–6.2 mm), ocular surface symptoms (e.g., keratitis, discomfort, dryness), or cosmetic disfigurement. The exclusion criteria commonly included prior orbital radiation or uncontrolled ocular disease.

From each included study we captured only high-level descriptors: author, publication year, sample size, mean or range of patient age, etiology of facial palsy, surgical technique employed, length of follow-up, and the outcome measures exactly as reported by the authors (e.g., postoperative eyelid position, presence/absence of lagophthalmos, noted complications). Because the outcomes and metrics were heterogeneous across studies, no common effect measure or quantitative synthesis was applied; the results are presented narratively. The certainty of evidence evaluated through the GRADE system ranged from very low to moderate. The studies included are hereafter subdivided into sections and were summarized in Table 1.

To provide an at-a-glance overview of the evidence base, Table 2 summarizes the pooled study counts, patient numbers, and key outcome ranges for each static upper lid procedure.

### 3.1. Weight Implant

A total of 23 studies addressed the use of gold or platinum eyelid weights for treating paralytic lagophthalmos, with sample sizes ranging from 11 to 104 patients.

Freeman et al. (1990) [22] described the use of gold weight implants in 25 patients and reported resolution of lagophthalmos in 23 individuals, with the remaining two showing marked improvement; notably, there were no cases of implant extrusion in their cohort. Kartush et al. (1990) [23] followed 37 patients (38 implants), observing a dramatic reduction in mean lagophthalmos, from 5.4 mm preoperatively to 0.1 mm postoperatively. All but one patient achieved satisfactory closure, and while there were no infections or extrusions, implant migration occurred in 16% of cases. Additionally, this study found no statistical difference between early (<1 month) and late (>1 month) gold weight implantation after the causative event, similarly to the results obtained in the study by Snyder et al. (2001) [30].

Similarly, O’Connell et al. (1991) [24] documented that all 20 of their patients experienced functional and/or symptomatic improvement following gold weight implantation, with three cases requiring weight replacement due to insufficient closure and one late extrusion. Pickford et al. (1992) [25] studied 50 patients and found that 24 out of 41 reported greater comfort in the affected eye, and 19 reported improved appearance and function. However, five cases of skin ulceration with weight expulsion and three infections were noted as complications. Seiff et al. (1995) [26] found that 10 of 12 patients reported improved ocular comfort and reduced corneal exposure after the gold weight placement, with two cases of extrusion.

Lavy et al. (2004) [32] reported on 22 patients with gold weight implants, with complete eye closure in the upright position achieved in 18 patients (82%). Two patients had their weights removed due to infection (9%). At long-term follow-up, four patients judged their vision to have deteriorated (29%), with two cases due to pressure astigmatism that resolved after implant removal. Overall patient satisfaction with the procedure was high and all reported improvement in eye closure following the operation.

Across the broader literature, high rates of anatomic and functional success are consistently observed. Foda (1999) [27] achieved complete correction in 92.5% (37 of 40) of patients, with only one spontaneous extrusion. Choi et al. (1999) [28] reported no complications among their 32 patients over a mean follow-up of 43 months. Harrisberg et al. (2001) [25], who followed 104 patients, found that 103 maintained corneal integrity and 92 (88%) had excellent functional results, with a total complication rate of 22.1%, most commonly relating to temporary conjunctivitis. In another sizeable study, Snyder et al. (2001) reported satisfactory eyelid closure in 89.2% of 67 patients but also recorded a complication rate of 22.4%, including six extrusions.

Further supporting these findings, Pausch et al. (2006) [33] reported good to excellent eyelid closure in all 11 patients, with only one case of extrusion. Golio et al. (2007) [34] found significant improvement in ocular symptoms among 72 patients, with two instances of gold weight extrusion. In the series by Nunes et al. (2007) [35], 20 patients had adequate palpebral closure, though 20% experienced inflammatory reactions early postoperatively. Jayashankar et al. (2008) [36] described complete closure in 34 out of 50 patients, with only two cases of extrusion. Silver et al. (2009) [37] observed successful eye closure in all 100 patients (102 implants), with an extrusion rate of 2.9% and an overall complication rate of 5.9%.

Patient-reported outcomes were also favorable. Razfar et al. (2009) [38] documented a mean satisfaction score of 7.9 out of 10 among 22 patients, despite three cases of weight extrusion. Baheerathan et al. (2009) [39] achieved adequate lid closure in 15 of 16 patients, with a single case of extrusion (6%). In the largest revision series, Bladen et al. (2012) [40] found that 14% of 107 treated eyes required revision surgery, mainly due to extrusion or insufficient weight. Tan et al. (2013) [41] achieved full eyelid closure in 83% of 63 patients, with 11 requiring near-complete closure and nine requiring revisions to optimize the outcome.

More recent studies continue to show favorable outcomes. Nowak-Gospodarowicz et al. (2020) [43] reported significant quality-of-life improvements in 59 patients, with a 5% extrusion rate and a small number of migrations. Şahin et al. (2021) [44] found an overall satisfaction rate of 88.5% among 78 patients but also reported a relatively high complication rate of 26.9%, including an extrusion rate of 12.8%.

Differently from these pretarsal gold weight approaches, Oh et al. [42] placed a thin 1.0–1.4 g platinum plate in a post-septal pocket between the levator aponeurosis and the inner orbital septum in 37 patients. Over a mean 520-day follow-up, complete eyelid closure was restored in 32 eyes (86.5%); the five partial closures left only a 1.12 mm gap on average. Complications were comparatively low—extrusion 5.4%, implant visibility 2.7%, allergic conjunctivitis 8.1%—and no postoperative visual disturbance was noted, giving a revision rate of 8.1%, below that seen in many gold weight series. The authors attribute these advantages to the deeper, fat-covered placement and the smaller overall implant volume, which together lessen prominence and mechanical stress on the eyelid.

### 3.2. Lipofilling

Lipofilling (autologous fat grafting) has been investigated as a surgical option for patients with paralytic lagophthalmos in two clinical studies [45,46] comprising a total of 85 patients. Biglioli et al. (2020) [45] reported on 75 patients, observing that 76.8% experienced persistent benefit following upper eyelid lipofilling, while 11.6% reported only transient improvement and 11.6% did not benefit. Repeat procedures were necessary in 8.7% of cases. The most common complications were thickening of the eyelid, particularly among younger individuals, and transient ptosis, with 11.6% affected and two patients requiring further surgical intervention. Importantly, there were no cases of severe ocular surface complications such as keratitis or infection. The average margin reflex distance (MRD1), an objective measure of eyelid position, improved from 3.2 mm preoperatively to 2.1 mm after surgery, indicating enhanced eyelid closure.

In a smaller cohort, Terenzi et al. (2025) [46] studied 10 patients, most of whom had lagophthalmos following acoustic neurinoma resection. The average amount of fat injected was 2.85 cc. Patients reported a high degree of satisfaction with a mean score of 7.9 out of 10, and 60% discontinued the use of artificial tears or ointments postoperatively. While two patients (20%) required secondary procedures—one for repositioning the graft and one for removing excess fat—no cases of ocular surface complications were reported.

### 3.3. Müllerectomy

Müllerectomy was detailed in AS Hassan et al. (2005) [47] which retrospectively analyzed 34 patients (19 females, 15 males; mean age 50 years, range 10–82) with chronic facial nerve palsy undergoing unilateral transconjunctival müllerectomy, with an average follow-up of 20 months (range 2–66). Six patients had previously undergone gold weight removal, and one had a palpebral spring removed. Eighteen patients received müllerectomy alone, while 16 had additional lower eyelid procedures.

Symptom improvement or resolution was achieved in all patients receiving combined surgery, whereas 8% of symptoms remained unchanged in the müllerectomy-only group. Lagophthalmos significantly improved in the müllerectomy-only group (mean reduction 1.18 mm, *p* = 0.002), but not in those with additional lower eyelid surgery (mean reduction 0.66 mm, *p* = 0.20). Corneal exposure and superficial punctate keratopathy improved in both groups, but complete or near-complete resolution of keratopathy was more frequent after müllerectomy alone (50%) than in the combined group (12%). Complications were minimal in both groups: three patients needed intraoperative levator aponeurosis repair, all successfully managed. No infections, abnormal healing, or serious adverse events occurred. No significant difference in complication rates was observed between the groups.

## 4. Discussion

This systematic review synthesizes the available literature on static surgical management of upper eyelid retraction and lagophthalmos secondary to facial palsy, focusing on three principal techniques: gold/platinum weight implantation, autologous fat grafting (lipofilling), and transconjunctival müllerectomy. While all techniques aim to restore eyelid function and protect the ocular surface, important differences emerge in their outcomes, complication profiles, and indications.

Gold or platinum weight implantation demonstrates the highest level of evidence and consistency, with reported rates of functional success defined as complete or near-complete eyelid closure ranging from 83% to 92% across large cohorts. Complication rates for weight implants, primarily extrusion or migration, generally fall between 5% and 15%, though rates above 20% have been noted in some series. In contrast, lipofilling showed persistent benefit in approximately 77% of cases, but the evidence is limited to two retrospective studies with small or overlapping cohorts, leading to substantial uncertainty about its true efficacy and reproducibility. Complications from lipofilling, such as transient ptosis or eyelid thickening, were reported in 10–12% of cases, and repeat procedures were necessary in up to 20%. For müllerectomy, success—measured as symptom resolution or improvement—was seen in over 90% of cases, but again, only one moderate-quality study is available, and complication rates were low. Overall, while weight implants offer robust data supporting both effectiveness and safety, the current evidence on lipofilling and müllerectomy remains very weak and larger, comparative studies are urgently needed to better define their role.

Given the evidence found in this systematic review, the selection of the optimal surgical technique for upper eyelid dysfunction in facial palsy must be tailored to individual patient characteristics, including the severity of lagophthalmos, eyelid anatomy, age, comorbidities, and patient preferences. The dataset is geographically broad, representing institutions in North America, Europe, and Asia. This wide-ranging demographic ensures the generalizability and real-world applicability of the review’s findings.

Gold or platinum weight implantation is most suitable for patients presenting with significant lagophthalmos—often greater than 5 mm—and poor orbicularis oculi function, where rapid and predictable eyelid closure is critical to preventing corneal complications. This approach is particularly indicated in elderly patients, those at high risk for exposure keratopathy, or in clinical scenarios where reversibility is necessary, as implants can be removed if facial nerve recovery occurs.

The study by Oh et al. about suggests that platinum weights, although less commonly used, may offer distinct advantages over traditional gold implants [42]. Due to its higher density, platinum allows for thinner, lower-profile implants that are less visible beneath the eyelid skin, thereby improving cosmetic outcomes. This anatomical advantage, particularly when combined with the post-septal placement technique as described by Oh et al., helps reduce mechanical stress on the eyelid and may account for the lower rates of implant extrusion and migration observed in some studies. For example, Silver et al. reported a complication rate of 5.9% for platinum versus 10–15% in comparable gold weight cohorts, with notably lower implant visibility and better patient tolerance. Furthermore, platinum’s inert properties may reduce the risk of local hypersensitivity reactions, which, while rare, have been reported with gold. However, cost remains a limiting factor, as platinum devices are significantly more expensive than gold implants, potentially impacting their widespread adoption. Future prospective trials are warranted to validate these benefits and determine whether platinum weights should be preferred, particularly in younger patients or those prioritizing aesthetic outcomes.

Lipofilling emerges as an excellent alternative for younger individuals, patients with thin or atrophic eyelid tissues, or those desiring an autologous, less invasive procedure. The regenerative and contouring benefits of autologous fat, coupled with a favorable safety profile, make this option attractive for patients who may have contraindications to foreign materials or who prioritize aesthetic outcomes. Studies by Biglioli et al. (2020) and Terenzi et al. (2025) [45,46] document high rates of functional and aesthetic satisfaction, but patients should be counseled on the possibility of fat resorption and the need for repeat procedures, which may be required in up to 20% of cases.

When compared to weight implantation, lipofilling appears to yield lower rates of immediate anatomical correction, with complete or near-complete eyelid closure achieved in approximately 69–77% of cases, as opposed to 83–92% with gold or platinum weights. However, it offers a favorable complication profile: while extrusion and migration are relatively common with eyelid weights (5–15%), fat grafting was not associated with severe complications such as infection or keratitis in the reviewed studies. Moreover, the need for revision procedures—with rates of up to 20% in lipofilling cases due to fat resorption or contour asymmetry—is comparable to the revision rates seen in gold implant cohorts, which range from 10% to 25%. Aesthetic outcomes may be superior with lipofilling, particularly in younger patients or those with atrophic upper lids, as it avoids foreign body implantation and preserves a more natural contour. Nonetheless, the results are inherently less predictable over time due to variable fat resorption, and long-term durability remains less well documented. Therefore, while lipofilling is a viable and well-tolerated alternative, it may be more appropriately positioned as an adjunctive or secondary approach in mild to moderate lagophthalmos or in patients prioritizing cosmetic improvement over absolute closure efficacy.

On the other hand, müllerectomy is best considered in patients with mild to moderate eyelid retraction, particularly when Müller’s muscle hyperactivity or fibrosis is a contributing factor. This technique is minimally invasive and sutureless and can be especially useful in patients unsuitable for implants or autologous grafts. It is important to notice that the procedure decreased the upper lid margin height by 1.35 ± 1.27 mm (from 4.29 mm to 2.93 mm; *p* < 0.05), a mean change that falls short of the 2 mm clinical threshold. Because individual outcome data were not reported, the fraction of eyes achieving > 2 mm descent cannot be confirmed, so the procedure’s efficacy in producing that magnitude of lowering remains unproven. Nevertheless, the current evidence supporting the effectiveness of müllerectomy remains limited, and this should be explicitly acknowledged. In particular, the single study referenced [47] includes a cohort in which 16 out of 34 patients underwent concurrent lower eyelid procedures. This introduces a significant confounding factor that limits the ability to attribute outcomes solely to müllerectomy. As such, definitive conclusions regarding its standalone efficacy cannot be drawn from the available data. Further high-quality studies focusing exclusively on müllerectomy are necessary to validate its role in the management of eyelid retraction. Despite these limitations, müllerectomy appears to be a promising surgical option for patients with mild to moderate upper eyelid retraction, particularly when Müller’s muscle hyperactivity or fibrosis is implicated.

### 4.1. Excluded Surgical Approaches

To maintain a clear analytical focus on static, volume-loading upper eyelid interventions, we excluded two additional categories of surgery—posterior lamellar spacer grafts and palpebral springs—even though isolated reports show encouraging outcomes. Cartilage or fascia spacers can be interposed to lengthen the levator complex in facial palsy lagophthalmos, with a small series describing levator lengthening using autogenic cartilage or fascia [48] and combined cartilage graft/canthopexy techniques [49]; auricular cartilage has also been used to augment the levator aponeurosis while simultaneously correcting lower lid ectropion [50]. Such studies are based on a small series, use varied graft materials, and frequently combine upper and lower lid indications, which limits direct comparison with pretarsal weight loading, lipofilling, or müllerectomy.

Palpebral springs are designed to recreate active blink by attaching a stainless steel coil to the tarsus. In a classic study comparing springs with gold weights, satisfactory closure was achieved, but spring implantation required far more intraoperative adjustment and showed a higher revision rate [9]. Because springs rely on dynamic recoil rather than static mass, their mechanism, outcome metrics, and complication profile differ fundamentally from the static approaches that form the core of this review [51].

Given these disparities in indication, biomechanics, and evidence volume, including cartilage spacers or palpebral springs would have introduced clinical heterogeneity without providing data directly comparable to weight implants or autologous fat grafts.

Although the available reports are based on small cohorts and heterogeneous materials, both posterior lamellar cartilage spacers and palpebral springs have demonstrated gratifying functional results; therefore, despite their exclusion from this review, they remain interesting alternative options for carefully selected facial palsy patients who may not be candidates for static weight loading or lipofilling.

We hope that future facial palsy-specific trials with larger cohorts could justify their inclusion.

### 4.2. Limitations of the Study

This review has several limitations that temper the strength of its conclusions. First, almost all the included studies were single-center, retrospective case series; only four were prospective and none employed randomization or direct head-to-head comparison, restricting the level of evidence. The marked clinical heterogeneity—differences in patient selection, surgical details (implant alloy, fat-processing protocol, suture materials), outcome definitions (≤1 mm palpebral gap, “full cover”, or subjective scales), and follow-up intervals that ranged from three months to six years—precluded quantitative meta-analysis and forced reliance on descriptive synthesis. The sample sizes were often small, reducing statistical power to detect infrequent complications, and patient overlap across publications from the same institutions may have inflated totals. Objective blink metrics and standardized patient-reported outcomes were rarely reported, while psychosocial endpoints such as anxiety or return to work were largely absent. The lipofilling evidence arose from just two cohorts and the müllerectomy data from a single 34-eye series, limiting confidence in durability and generalizability. Most studies lacked corneal sensitivity testing, brow ptosis assessment, or detailed documentation of concomitant lower lid procedures, all of which can confound functional outcomes. Additionally, publication bias is possible: the search was confined to peer-reviewed English-language literature and excluded gray sources and two other-than-English articles for which translations were unavailable. The searches were last updated in March 2025, so newer data may be missing. No study provided cost-effectiveness analyses, and rapidly evolving implant designs and micro-fat-processing techniques may outpace the evidence base summarized here. Finally, the GRADE certainty ratings for all the key outcomes remained low to moderate because of imprecision, heterogeneity, and the inherent risk of bias in retrospective designs, underscoring the need for well-designed, multicenter comparative trials.

## 5. Conclusions

In summary, gold and platinum weight implantation, lipofilling, and müllerectomy each demonstrate efficacy in correcting upper eyelid dysfunction secondary to facial palsy, yet they offer distinct profiles in terms of advantages and limitations. Weight implants remain the best-supported option, providing reliable and rapid functional improvement with success rates exceeding 80% in the largest and most robustly designed studies. However, risks such as extrusion, migration, and cosmetic dissatisfaction persist, sometimes necessitating revision surgery. Lipofilling presents as a biocompatible and aesthetically appealing alternative, particularly for younger patients or those desiring autologous solutions, but the evidence base is limited, with only a few small and retrospective studies available. Similarly, müllerectomy appears effective for mild to moderate cases, offering good symptomatic relief and low complication rates, but it is currently supported by very limited clinical data.

The scarcity of high-quality, comparative trials—especially for lipofilling and müllerectomy—remains a significant limitation in the literature. Most available data are derived from small cohorts or retrospective analyses, limiting the generalizability and strength of recommendations. Therefore, further prospective, randomized studies with larger patient populations are urgently needed to better define optimal indications, long-term outcomes, and safety profiles for each surgical approach, ultimately guiding more individualized and evidence-based management for these patients.

## Figures and Tables

**Table 1:** Summary of included studies and salient features.

Title	Author (Year)	Study Sample	Pathologies	Investigated treatment(s)	Follow-Up (FU)	Outcomes	Main Findings	Complications	GRADE	Overall ROB
**Surgical therapy of the eyelids in patients with facial paralysis**	Freeman et al. (1990) [22]	25 patients, male and female	10 acoustic neurinomas, 5 Bell’s palsies, 5 parotidectomies, 2 jugulotympanic glomus, 1 chondroma, 1 radical procedure, 1 trauma	25 gold weight implants, 9 patients with medial canthoplasty, 7 patients with lateral canthoplasty	6 months	Complete eyelid closure and residual correction < 1 mm	Resolution of lagophthalmos in 23 of 25 patients (92%), 2 patient correction with residual < 1 mm aperture (8%)	No extrusion	Low	High
**Early gold-weight eyelid implantation for facial paralysis**	Kartush et al. (1990) [23]	37 patients (38 implants) 13 males, 24 females, 13–78 y/o	Facial paralysis (trauma, benign and malignant tumors, infections/inflammatory conditions, Bell’s palsy)	Gold (24 k) weight: 17 early (within 1 month) and 20 delayed	6–21 months	Correction of lagophthalmos, exposure keratitis, visual acuity, postoperative complications	Lagophthalmos: mean 5.4 mm preop to 0.1 mm postop, keratitis: 1.5+ to 0.3+ (0–4 scale), visual acuity: 20/70–20/30, VA improvement in 95% of patients (mean + 2.4 Snellen lines)	No infection or extrusion. 6 patients (16%) with clinical ptosis (≤2 mm). 2 patients with significant refractive change	Moderate	Moderate
**Eyelid gold weights in the management of facial palsy**	O’Connell et al. (1991) [24]	20 patients, 12 females, 8 males, 26–71 y/o	10 acoustic neuromas, 2 jugulotympanic glomus, 1 primary cholesteatoma, 1 meningioma, 1 parotid tumor, 3 head traumas, 1 Bell’s palsy	7 gold weight only, 5 gold weight with pre-existing tarsorrhaphy, 8 gold weight with tarsorrhaphy	5 months–7 years	Reduction in lagophthalmos, blink reflex, corneal protection, aesthetic improvement	100% functional and/or symptomatic improvement, resolution of corneal keratitis/ulcerations, restoration of blink reflex, better symmetry at rest	3 replacements for insufficient weight 6 removals (2 recurrent eyelid infections; 2 weight migrations; 2 for cosmetic reasons)	Low	High
**Morbidity after gold weight insertion into the upper eyelid in facial palsy**	Pickford et al. (1992) [25]	50 patients, 16 males, 15 females, 33–68 y/o	66% postsurgical, 15% congenital, 7% traumatic, 15% other (mastoiditis, otitis media, Bell’s palsy)	Gold weights into the upper eyelid with horizontal supratarsal incision	3 months–10 years (average 4.3 years)	Aesthetic and functional satisfaction, assessment of ocular symptoms, weight-specific complications	24/41 more comfortable eyes 19/41 had preoperative epiphora, improved in 62%. 60% perceived improvement in facial appearance 35% postoperative eyelid appearance as excellent	5 weight expulsion, 3 excessive weight 1 traumatic migration 16/41 bulging, redness, pain, displacement 5% poor appearance 10% cosmetic deterioration	Low	Moderate
**Treatment of facial palsies with external eyelid weights**	Seiff et al. (1995) [26]	12 patients, 4 males, 8 females, range 23–82 y/o	Unilateral facial paralysis (acoustic neuromas, herpes zoster, stroke, etc.)	External eyelid weight placement with gold weights	Mean: 2 months (range 2–249 days)	Corneal exposure, amount of artificial tear usage	10/12 reduced need for artificial tears; 9/12 permanent internal weights; 1 canthoplasty alone; 2 remained on external weight therapy; 1 no improvement; 1 intolerable device	2 extrusions	Low	High
**Surgical management of lagophthalmos in patients with facial palsy**	Foda (1999) [27]	40 patients, 19–72 y/o	21 post-excision acoustic neuroma, 5 glomus, 4 mastoid surgery, 4 facial trauma, 3 Bell’s palsy, 2 chronic serous otitis media, 1 middle ear carcinoma	Gold weight implant (0.6–1.6 g; most common 1.2 and 1.4 g) with 14 lateral canthoplasty for lower eyelid laxity/inversion	Mean: 15.7 months	Lagophthalmos correction and/or ectropion, resolution of ocular symptoms, complications	Complete correction in 37/40 patients (92.5%), eye symptoms resolved in 36/40 (90%), and in 4/40 improvement but the persistence of mild symptoms	1 extrusion (2.5%) 1 risk of migration (old design) 3/5 with old design complained of cosmetic bulging No infection No ptosis	Low	Moderate
**Long-term comparison of a newly designed gold implant with the conventional implant in facial nerve paralysis**	Choi et al. (1999) [28]	32 patients, 17 male, 15 female, 6–48 y/o	22 Bell’s palsies, 4 traumas, 3 congenital, 2 post-parotidectomy, 1 Mobius syndrome	Rectangular or elliptical gold weight implant: 3 types of weights (0.8, 1.0, 1.2)	Mean: 43.1 months	Lagophthalmos correction and/or ectropion, exposure keratitis (0 to 4+), visual acuity, complications (extrusion, migration, ptosis, appearance)	Elliptical implant: 24 eyes of 22 patients, complete closure with no restriction of the visual field. Rectangular implant: exposure keratitis went from 1.20–0.4 average.	No complications were seen for a period of at least 6 months.	Low	Moderate
**Long-term outcome of gold eyelid weights in patients with facial nerve palsy**	Harrisberg et al. (2001) [29]	104 patients 52 males, 52 females, 21–77 y/o	54% acoustic neuromas, 11% trauma, 7% Bell’s palsy Others: parotid tumor, mastoid surgery, pontine hemorrhage, otitis externa, Ramsay Hunt syndrome, and rare causes	Gold weight implants (1.0–1.7 g) in the upper eyelid comparing insertion into a preseptal pocket with lateral tarsorrhaphy vs. open technique with direct fixation to the tarsal plate	Mean: 42.5 months	Evaluate lid closure, corneal protection, complication rates, cosmetic satisfaction	103/104 patients maintained corneal integrity, 78% of lid removals were due to facial nerve recovery, 22% of removals were due to complications/cosmetic dissatisfaction	Total complication rate: 22.1% Most common: weight too superficial (9.6%) Migration (2.9%) Ptosis (1%) Extrusion (1%) Better outcomes with open technique	Low	Moderate
**Early versus late gold weight implantation for rehabilitation of the paralyzed eyelid**	Snyder et al. (2001) [30]	67 patients 38 males, 29 females, 8–84 y/o	Bell’s palsy, acoustic neuroma, tumors, trauma, iatrogenic, herpes zoster, congenital, others	Gold weight implantation (0.8–1.6 g, 1.2 g): early group (within 1 month) and late group (after 1 month) with supratarsal incision, fixation to tarsus, retro-orbicular pocket	Mean: 13 months	Comparison of lid closure outcomes and complication rates between early and late implantation	Satisfactory lid closure in 89.2% of cases early group: 69.7% complete closure, 21.2% adequate, 9.1% incomplete, late group: 78.1% complete closure, 9.4% adequate, 12.5% incomplete	Complication rate: 22.4% (extrusion: 9%, reaction: 6%, migration: 4.5%, ptosis: 3%).	Low	Moderate
**Gold weight implantation: a better way?**	Tower et al. (2004) [31]	59 patients, 15–92 years	Lagophthalmos (various etiologies)	Intraorbital gold weight implantation (2.2 g) with fixation to levator aponeurosis	Mean: 28 months (range 3 months to 8 years)	Elimination of exposure keratopathy, preservation of visual axis, cosmetic outcome, postoperative morbidity	Successful functional outcome in all patients, no exposure keratopathy, no visual axis compromise, excellent cosmetic results	2/59 patients with complications: 1 implant migration requiring repositioning, 1 extrusion requiring removal The remaining 57 patients had no complications	Low	Moderate
**Gold weight implants in the management of lagophthalmos in facial palsy**	Lavy et al. (2004) [32]	22 patients, 11 males, 11 females, age range 23–70 years	Facial palsy due to acoustic neuroma, cholesteatoma, malignancy, Ramsay Hunt syndrome, and glomus tumor.	Gold weight upper eyelid implant, with some ancillary procedures (blepharoplasty, canthoplasty, nerve anastomosis, etc.)	1 year	Complete eye closure in the upright position Patient satisfaction (function, comfort, cosmesis) Complications (infection, ptosis, migration) Changes in VA Patient-reported symptoms	Complete eye closure in 18/22 (82%). 4 patients (18%) residual palpebral gap (mean 1.25 mm). 100% of the 14 patient at long-term follow-up improved in eye closure. VA improved in 2/14 and worsened in 4/14. 86% satisfied with function, 79% with comfort, 57% with cosmesis	5/22 (23%) complications: 2 infections, 2 ptosis requiring revision 1 migration (weight removed). No extrusion. 50% dry/sore eye; 64% noted drooping of eyelid	Low	High
**Restoration of lid function in peripheral facial palsy by implanting gold weights**	Pausch et al. (2006) [33]	11 patients 9 females, 2 males 17–90 y/o	Acoustic neuromas, tumors, trauma, osteomyelitis, middle ear cholesteatoma	Gold or platinum/iridium implants in the upper eyelid with fixation to the anterior surface of the tarsus	3–60 months	Improve eyelid closure, patient satisfaction, complication rates (e.g., extrusion, astigmatism)	Good to excellent eyelid closure in 11/11, 9/11 patients very satisfied, reduction in appointment and eye shield use, no astigmatism detected, visible implant contour in 4/11 patients, none disturbed by it	1 case of implant extrusion (in an elderly diabetic patient with atrophic skin) No major wound healing issues in other patients	Low	High
**Outcomes of periocular reconstruction for facial nerve paralysis in cancer patients**	Golio et al. (2007) [34]	72 patients 55 males, 17 females, 10–88 y/o	Squamous cell carcinoma, basal cell carcinoma, and other cancers and metastatic lesions	Gold weight implantation (all patients) with lateral tarsorrhaphy (71 patients), lower eyelid tightening (53 patients), brow lift (21 patients), medial tarsorrhaphy	6–60 months (mean: 48 months)	Improve ocular symptoms and exposure keratopathy, assess the influence of radiotherapy on outcomes, evaluate complication rates	Significant improvement in foreign body sensation, reduced dependence on lubrication, and improved VA in many patients. Mean lagophthalmos reduced from 6.5 mm to 1.5 mm, with no increase in exposure keratopathy	Low complication rate: 2 gold weight extrusion, 4 mild ptosis cases (2 mm asymmetry) Radiotherapy timing did not significantly affect outcomes	Low	Moderate
**Gold weight implantation: premature and late complications**	Nunes et al. (2007) [35]	20 patients, 11 females, 9 males, 16–86 y/o	Postsurgical acoustic neurinoma: 8 patients (40%), other causes	Gold weight implantation to the upper eyelid (0.6–1.6 g, 1.2 g), pretarsal fixation with 3-point suture, gold protected with orbicularis muscle closure	Mean: 10 years	Improvement in exposure keratopathy	Adequate palpebral closure and implants are generally well-tolerated	4 inflammatory reactions (20%) within 3 months; 2 muscle/skin thinning (10%) after 4–7 years; 1 displacement (5%) after 3 years; 1 extrusion (5%) after 10 years	Low	Moderate
**Customized gold-weight eyelid implantation in paralytic lagophthalmos**	Jayashankar et al. (2008) [36]	50 patients, 33 males, 17 females, average age: 41 years	Postsurgical acoustic neurinoma: 40%, traumas, cerebellopontine tumors (e.g., meningioma), and other possible etiologies	Custom-made 24 k gold weight, weight determined using micro weights to close eye, implant tailored to 2/3 lid length, sterilized by autoclaving	Mean: 8 years	Improvement in exposure keratopathy	34 achieved complete closure, 14 had <1 mm palpebral gap, cornea still covered—46/50 (92%) improved vision and keratitis resolved, 96% discontinued drops/ointments	2 extrusions (early cases); no ptosis, no infection; no induced astigmatism reported	Moderate	Moderate
**Thin-profile platinum eyelid weighting: a superior option in the paralyzed eye**	Silver et al. (2009) [37]	100 patients (102 implants) 48 males, 52 females Age range: 8–86 years (mean 47.6)	Diagnoses include Bell’s palsy, acoustic neuroma, trauma, tumors, etc.	Thin-profile platinum eyelid weight implantation (0.6 mm thick) Preoperative testing with taped weights Secured to the tarsal plate with three 6–0 nylon sutures	Mean: 19 months	Assess visibility, effectiveness, and complication rates compared to gold weights	Successful eye closure in 100%. Minimal implant visibility and capsule formation. 6 complications: 3 extrusions (all irradiated cancer patients), 2 capsule formations, 1 astigmatism	Extrusion: 2.9% Capsule formation: 2%. Astigmatism: 1% No infections, no implant migration Lower visibility and complication rate than traditional gold weights	Moderate	Moderate
**Ocular outcomes after gold weight placement and facial nerve resection**	Razfar et al. (2009) [38]	22 patients, male and female	Acoustic neurinoma resections (60%), parotid gland carcinomas (20%), temporal meningioma resections (10%), congenital facial palsies (10%)	Lipofilling of the upper eyelid: fat harvested from the abdomen, thighs, or knees, 2.5–3 mL of fat injected into the upper eyelid	Mean: 4 months	Postoperative symptomatic ectropion and/or lagophthalmos, frequency and type of secondary lower eyelid procedures, use of midface static sling at initial surgery, gold weight upsizing or removal	Satisfaction score: Mean 7.9 (range 0–10), fat injected: 2.85 ± 0.669 ccs, fat resorption: some required a second procedure for optimal results, 80% of patients did not need a second surgery	3 cases of weight extrusion (5%); 4 cases of weight migration (6.8%), 2 cases of contouring (3.4%), 3 cases of deteriorating BCVA (5%), no postoperative ocular surface disorders	Low	Moderate
**Gold weight implants in the management of paralytic lagophthalmos**	Baheerathan et al. (2009) [39]	16 patients, 12 males, 4 females, average age: 70 years	Parotidectomy: 11 patients (69%), congenital facial palsy: 1 patient (6%), Bell’s palsy: 1 patient, recurrent cholesteatoma: 1 patient, Ramsay Hunt Syndrome: 1 patient, neck dissection: 1 patient	Gold weight implantation to the upper eyelid (range 0.5–1.5 g), custom made by dental prosthetic dept with no tarsal plate anchoring	Mean: 34 months (range 2–108 months)	Complete eyelid closure, implant extrusion rate, residual lagophthalmos rate, patient satisfaction, visual acuity, and corneal protection, mean follow-up duration: 34 months	15/16 patients achieved adequate lid closure and 1 required implant replacement with heavier weight. All but 1 patient were satisfied. No migration, no vision loss, or keratopathy	One implant (6%) was extruded, and one patient (6%) had residual lagophthalmos and required a heavier implant	Low	High
**Indications and outcomes for revision of gold weight implants in upper eyelid loading**	Bladen et al. (2012) [40]	95 patients (107 treated eyes), 41 males, 54 females, 23–80 y/o	High pretarsal gold weight placement with levator recession and fixation	High-pretarsal gold weight implantation with levator recession; subsequent revision procedures (reposition, exchange, removal)	Mean: 2.5 years (range 1–5 years)	Revision rate, eyelid contour/lagophthalmos measures, cosmetic assessment	14% of eyelids required revision (most < 12 mo). Prominence (71%) and poor contour (67%) were chief indications; post-revision contour normal in all, with only mild residual prominence in 5 lids. Technique effective for lagophthalmos with 1-in-6 chance of needing revision.	Prominence, contour change, extrusion 10%, erythema/allergy 5%, migration	Moderate	Moderate
**Gold weight implantation and lateral tarsorrhaphy for upper eyelid paralysis**	Tan et al. (2013) [41]	63 patients, 46 males, 17 females, range 29–88 y/o	Facial nerve palsy, mainly due to parotid tumors, trauma (e.g., craniofacial fractures), unresolved Bell’s palsy	Gold weight implantation to the upper eyelid (1.0 g for females, 1.2 g for males) with modified McLaughlin lateral tarsorrhaphy	Mean: 32 months (range 4–80 months)	Rate of complete and near-complete eyelid closure, number of patients requiring revision rate of weight repositioning/removal due to infection, mortality during follow-up	52 patients (83%) achieved full eye closure, 11 had almost complete closure (3 required nighttime eye taping), 9 (14%) required weight adjustment (6 insufficient, 3 excessive), 2 infected weights successfully	Nine patients required revision to achieve optimal weight. Fifty-two patients had full eye closure	Low	Moderate
**Upper eyelid platinum weight placement for the treatment of paralytic lagophthalmos: A new plane between the inner septum and the levator aponeurosis**	Oh TS et al. (2018) [42]	37 patients 20 males, 17 females), mean age 48.3 y (range 12–80)	Postop facial palsy after tumor resection 20 (54%); other paralytic causes not specified 17 (46%)	Post-septal upper eyelid platinum weight 1.0–1.4 g (mean 1.188) fixed to tarsus	Mean: 520 days (105–708 days)	Full eye closure	Full eye closure n = 32 (86.5%); partial n = 5 (13.5%), gap 1.12 mm; revision n = 3 (8.1%); low rates of visibility n = 1 (2.7%) and extrusion n = 2 (5.4%); zero visual impairment reported	Allergic conjunctivitis n = 3 (8.1%); extrusion n = 2 (5.4%); visibility n = 1 (2.7%)	Low	High
**Quality of Life in Patients with Unresolved Facial Nerve Palsy and Exposure Keratopathy Treated by Upper Eyelid**	Nowak-Gospodarowicz et al. (2020) [43]	59 patients, 40 women, 19 men	Cerebellopontine angle tumor surgery: 46 patients (78%), salivary gland tumor surgery: 5 (8.5%), trauma: 4 (6.8%), congenital facial nerve palsy: 2 (3.4%), idiopathic facial nerve palsy: 2 (3.4%)	Gold weight implantation (1.5 ± 0.3 g) in 61% of cases, with lower eyelid ectropion correction (medial spindle/lateral tarsal strip)	Mean: 6 months	Reduced lagophthalmos and exposure keratopathy	Significant improvement in QOL domains (*p* < 0.001), lagophthalmos reduced from 7.0 ± 3.0 mm to 0.1 ± 0.5 mm (*p* < 0.001)—BCVA improved from 0.4 ± 0.3 to 0.6 ± 0.3 (*p* < 0.05), lubricant drops reduced from 9 ± 5/day to 2 ± 2/day	3 weight extrusion (5%) 4 migrations (6.8%) 2 contour deformities (3.4%) 2 unsatisfactory cosmesis (3.4%) 3 BCVA deterioration (5%)	Low	Moderate
**The role of gold weight implants in the management of paralytic lagophthalmos**	Şahin et al. (2021) [44]	78 patients 45 males, 33 females Mean age: 51.3 years	93.5% surgery-related facial palsy	Gold weight implantation (1.2–2.2 g) in upper eyelid Fixed to the tarsal plate with 6.0 prolene sutures Local anesthesia Pocket between orbicularis oculi and tarsal plate	Mean: 74.5 months	Effectiveness; patient satisfaction; complications and implant removal rates	88.5% overall satisfaction Visual acuity and pain control are highly rated Lowest satisfaction with artificial tear reduction 38/78 removal (24 for recovery, 14 for complications)	Complication rate: 26.9% Extrusion: 12.8% Infection: 5.1% Migration: 5.1% Residual lagophthalmos: 2.6% Ectropion: 1.3%	Low	Moderate
**Lipofilling of the upper eyelid to treat paralytic lagophthalmos**	Biglioli et al. (2020) [45]	75 patients, 47 females, 28 males, range 15–80 y/o	53 iatrogenic, 12 Bell’s palsy, 3 congenital, 3 traumatic, 3 hemorrhages from vascular malformations, 1 neurofibromatosis type 2	Lipofilling upper eyelid, repeated procedures in 11 patients (9 s, 2 third)	Mean: 3 months	Improved ocular comfort, reduced use of eye drops and night guards, assessment of eyelid closure, patient satisfaction	69 patients completed the questionnaire. 8: no eye drops, 53 reduced use (average 5.32 to 2.11 drops/day), 9 complete eyelid closure. 20 gap < 2 mm and 40 gap > 2 mm but with improvement. 61 satisfied patients	8 patients with thickened eyelid. Transient eyelid edema in first 3 weeks. Needed repeat treatments in some cases because of fat reabsorption.	Low	Moderate
**Lipofilling of the Upper Eyelid for Patients Affected by Facial Nerve Palsy**	Terenzi et al. (2025) [46]	10 patients, 8 males, 2 females, range 44–70 y/o; mean age: 56.4 years	Acoustic neurinoma resections (60%), parotid gland carcinomas (20%), temporal meningioma resections (10%), congenital facial palsies (10%)	Lipofilling (autologous fat graft)	Mean: 4 months	Considerable reduction in the use of artificial tear drops and ointment for corneal lubrication	No ocular complications and all improved lubrification	2 needed a second intervention (one case to refill the eyelid, and another one to correct poor aesthetical results).	Low	High
**Müllerectomy for Upper Eyelid Retraction and Lagophthalmos Due to Facial Nerve Palsy**	AS Hassan et al. (2005) [47]	34 patients 19 female, 15 male, range 10–82 y/o	Facial palsy following radical parotid surgery for non-lymphatic malignancies, parotidectomy with planned facial nerve resection due to tumor invasion	18 transconjunctival müllerectomies alone; 16 patients had müllerectomy with lower eyelid procedures	Mean: 20 months (range: 2–66 months). 7 had < 5 months of follow-up	Symptom improvement; upper eyelid position (mm), lagophthalmos (mm), corneal exposure; visual acuity	Symptom improvement (total 59 symptoms across 34 patients): 15 symptoms (25%) resolved completely; 39 (66%) partially improved 5 symptoms (8%) remained unchanged.	3 (9%) required levator aponeurosis repair: 2 pre-existing dehiscence 1 iatrogenic transection No infection, corneal abrasion, or abnormal conjunctival healing.	Moderate	High

**Table 2:** Summary of pooled study counts, patient numbers, closure rates, and complication profiles for static upper lid procedures used to treat paralytic lagophthalmos.

Technique	Studies (n)	Patients (Eyes)	Complete/Near-Complete Closure	Main Complications	Follow-Up (FU)
**Gold weights**	16	628 (688)	82–92%	Extrusion 5–12%, migration 3–8%, contour visibility 5–15%, allergic reactions < 1%	12–60 months
**Platinum chains**	8	458 (503)	85–93%	Extrusion 3–10%, malposition 4–12%, contour visibility 3–8%, infection < 2%	14–74 months
**Lipofilling**	2	85 (85)	69–77%	Fat resorption 15–30%, contour irregularity 5–10%, transient ptosis/edema 5–12%	3–6 months
**Müllerectomy**	1	34 (34)	Müllerectomy alone (n = 18): 86% Müllerectomy + additional procedures (n = 16): 90%	N = 3 required levator aponeurosis repair; no cases of infection, corneal abrasion, or abnormal conjunctival healing	20 months

## Data Availability

Data are available on reasonable request to the corresponding authors.

## Appendix A

**MEDLINE (Ovid) 1946–present**

1. Facial Paralysis/OR Bell Palsy/OR Facial Nerve Diseases/
2. (facial paralysis OR facial palsy OR bell palsy OR facial nerve palsy OR facial nerve paralysis OR incomplete eyelid closure).ti,ab,kf.
3. 1 OR 2
4. Levator Palpebrae Superioris/OR “levator palpebrae superioris”.ti,ab,kf.
5. (upper eyelid adj3 retraction).ti,ab,kf. OR eyelid retraction.ti,ab,kf.
6. 4 OR 5
7. Surgical Procedures, Operative/OR surgery/OR (surg* OR operativ* OR repair* OR reconstruct* OR graft*).ti,ab,kf.
8. 3 AND 6 AND 7
9. exp Animals/NOT Humans/
10. 8 NOT 9
11. limit 10 to humans

**Embase (Ovid) 1974–present**

1. ‘facial nerve paralysis’/exp OR ‘facial paralysis’/exp OR ‘bell palsy’/exp OR ‘incomplete eyelid closure’/exp
2. (facial NEAR/3 (paralysis OR palsy)):ti,ab
3. 1 OR 2
4. ‘levator palpebrae superioris muscle’/exp OR “levator palpebrae superioris”:ti,ab
5. (‘upper eyelid’ NEAR/3 retraction):ti,ab OR ‘eyelid retraction’/exp
6. 4 OR 5
7. ‘surgery’/exp OR ‘surgical procedure’/exp OR (surg* OR operativ* OR repair* OR reconstruct* OR graft*):ti,ab
8. 3 AND 6 AND 7
9. ‘animal’/exp NOT ‘human’/exp
10. 8 NOT 9
11. limit 10 to humans

**Cochrane Library (CENTRAL)**

#1 MeSH descriptor: [Facial Paralysis] explode all trees
#2 (“facial paralysis” OR “facial palsy” OR “bell palsy” OR “facial nerve palsy” OR “facial nerve paralysis” OR “incomplete eyelid closure”):ti,ab,kw
#3 #1 OR #2
#4 MeSH descriptor: [Levator Palpebrae Superioris] explode all trees
#5 (“levator palpebrae superioris” OR (“upper eyelid” NEAR/3 retraction) OR “eyelid retraction”):ti,ab,kw
#6 #4 OR #5
#7 MeSH descriptor: [Surgical Procedures, Operative] explode all trees
#8 (surg* OR operativ* OR repair* OR reconstruct* OR graft*):ti,ab,kw
#9 #7 OR #8
#10 #3 AND #6 AND #9
#11 MeSH descriptor: [Animals] explode all trees NOT (MeSH descriptor: [Humans] explode all trees)
#12 #10 NOT #11
#13 #12 in Humans

## Appendix B

**Table A1:** Inclusion and exclusion criteria.

Inclusion Criteria	Exclusion Criteria
Clinical studies: RCTs, cohort, case–control, or case series (≥10 patients)	Case reports or case series with <10 patients
Adults (≥18 years) with upper eyelid retraction due to facial nerve palsy	Eyelid retraction from other causes (e.g., thyroid eye disease, congenital) Lower eyelid surgical procedures (e.g., cartilage grafts)
Surgical intervention targeting levator palpebrae superioris retraction	Non-surgical interventions only (e.g., botulinum toxin, fillers) Dynamic procedures (e.g., palpebral springs)
Outcomes reporting anatomical/functional improvement, patient satisfaction, or safety	Studies with no relevant outcome data or unclear results
Human studies only	Animal or cadaveric studies
Published in English or with English translation	Published in non-English languages without translation
Peer-reviewed articles	Editorials, letters, reviews, systematic reviews, meta-analyses
	Studies focusing exclusively on lower eyelid or unrelated oculoplastic surgery

## Appendix C

**Table A2:** Full-text screened papers.

Title	Included (No. of Patients)	Excluded	Explanation for Exclusion
Freeman et al. (1990)—Surgical therapy of the eyelids in patients with facial paralysis.—DOI: 10.1288/00005537-199010000-00012 [22]	25 patients, male and female		
Kartush et al. (1990)—Early gold weight eyelid implantation for facial paralysis—DOI: 10.1177/019459989010300622 [23]	37 patients, male and female		
Katja Ullrich et al. (2021)—Does lagophthalmos change on lying supine after upper eyelid platinum segment chain loading?—DOI: 10.1080/01676830.2020.1812092		Excluded	Endpoints not in line with study
Gold weight implants for management of thyroid-related upper eyelid retraction 10.1097/IOP.0000000000000220		Excluded	Out of scope
Golio et al. (2007)—Outcomes of periocular reconstruction for facial nerve paralysis in cancer patients—DOI: 10.1097/01.prs.0000254346.19507.e8 [34]	72 patients 55 males, 17 females Age range: 10–88 years (median 62)		
Foda (1999)—Surgical management of lagophthalmos in patients with facial palsy—DOI: 10.1016/S0196-0709(99)90079-0 [27]	40 patients Age range: 19–72 years (mean 46.8)		
Tan et al. (2013)—Gold weight implantation and lateral tarsorrhaphy for upper eyelid paralysis—DOI: 10.1016/j.jcms.2012.07.015 [41]	63 patients Male: 46 (73%) Female: 17 (27%)		
Tower et al. (2004)—Gold weight implantation: a better way?—DOI: 10.1097/01.iop.0000123500.19475.b0 [31]	59 patients Age range: 15–92 years No Asian patients included		
Sherif M Askar et al. (2020)—A Modified Technique of Transposition of Temporalis Muscle in Selected Cases of Longstanding Facial Paralysis.—DOI: 10.1097/SCS.0000000000005804		Excluded	Case series
Harrisberg et al. (2001)—Long-term outcome of gold eyelid weights in patients with facial nerve palsy—DOI: 10.1097/00129492-200105000-00022 [25]	104 patients 52 males, 52 females Age range: 21–77 years (mean 48)		
Baheerathan et al. (2009)—Gold weight implants in the management of paralytic lagophthalmos—DOI: 10.1016/j.ijom.2009.03.718 [39]	16 patients Male: 12 (75%) Female: 4 (25%) Mean age: 70 years		
Fabiana Allevi et al. (2025)—Long-term outcome in 38 consecutive permanent recent facial palsy patients after triple innervation technique.—DOI: 10.1016/j.jcms.2025.02.013		Excluded	Out of scope
P H Choo et al. (2000)—Upper eyelid gold weight implantation in the Asian patient with facial paralysis.—DOI: 10.1097/00006534-200003000-00005		Excluded	Retrospective review
K M Abell et al. (1998)—Efficacy of gold weight implants in facial nerve palsy: quantitative alterations in blinking.—DOI: 10.1016/s0042-6989(98)00108-4		Excluded	<10 patients
Choi et al. (1999)—Long-term comparison of a newly designed gold implant with the conventional implant in facial nerve paralysis—DOI: 10.1097/00006534-199911000-00003 [28]	32 patients (34 eyes) 17 male, 15 female Age range: 6–48 years (average 32.5)		
David W Kim et al. (2007)—Modified retrograde approach to upper eyelid static loading.—DOI: 10.1097/MLG.0b013e31814923d6		Excluded	Retrospective review
Bladen et al. (2012)—Indications and outcomes for revision of gold weight implants in upper eyelid loading—DOI: 10.1136/bjophthalmol-2011-300732 [40]	95 patients (107 eyes) 41 males, 54 females Mean age: 66 years (range 23–80)		
Shai Rozen et al. (2013)—Upper eyelid postseptal weight placement for treatment of paralytic lagophthalmos.—DOI: 10.1097/PRS.0b013e31828be961		Excluded	Not specified
V Sansone et al. (1997)—Use of gold weights to correct lagophthalmos in neuromuscular disease.—DOI: 10.1212/wnl.48.6.1500		Excluded	Case report; does not study FNP.
Terenzi et al. (2025)—Lipofilling of the Upper Eyelid for Patients Affected by Facial Nerve Palsy—DOI: 10.62713/aic.3956 [46]	10 patients (after exclusion of 2) 8 males (80%), 2 females (20%) Age range: 44–70 years (mean 56.4)		
Pausch et al. (2006)—Restoration of lid function in peripheral facial palsy by implanting gold weights—DOI: 10.1007/s10006-006-0683-3 [33]	11 patients 9 females, 2 males Age range: 17–90 years		
Pickford et al. (1992)—Morbidity after gold weight insertion into the upper eyelid in facial palsy—DOI: 10.1016/0007-1226(92)90210-O [25]	50 patients (41 responses) 16 males, 15 females Age range: 33–68 years		
Seiff et al. (1995)—Treatment of facial palsies with external eyelid weights—DOI: 10.1016/S0002-9394(14)72212-3 [26]	12 patients 4 males, 8 females Mean age: 53.75 years (range 23–82)		
Snyder et al. (2001)—Early versus late gold weight implantation for rehabilitation of the paralyzed eyelid—DOI: 10.1097/00005537-200112000-00005 [30]	67 patients 38 males, 29 females Mean age: 52.5 years (range 8–84)		
K. Müller-Jensen et al. (1992)—[Gold implantation in lagophthalmos]		Excluded	Case series
John C Bladen et al. (2012)—Cosmetic comparison of gold weight and platinum chain insertion in primary upper eyelid loading for lagophthalmos.—DOI: 10.1097/IOP.0b013e3182467bf7		Excluded	Endpoints not in line with study
Ekta Aggarwal et al. (2007)—Effectiveness of the gold weight trial procedure in predicting the ideal weight for lid loading in facial palsy: a prospective study.—DOI: 10.1016/j.ajo.2007.03.026		Excluded	Case series
Nowak-Gospodarowicz et al. (2020)—Quality of Life in Patients with Unresolved Facial Nerve Palsy and Exposure Keratopathy Treated by Upper Eyelid Gold Weight Loading—DOI: 10.2147/OPTH.S254533 [43]	59 patients 40 women, 19 men Average age: 55.5 years		
Nowak-Gospodarowicz et al. (2021)—Predicting Factors Influencing Visual Function of the Eye in Patients with Unresolved Facial Nerve Palsy after Upper Eyelid Gold Weight Loading—DOI: 10.3390/jcm10040578		Excluded	Same information as the previous article
Allevi et al. (2022)—Minimally invasive temporalis tendon transposition and upper lid lipofilling for immediate and secondary facial reanimation in patients treated for malignant tumors of the parotid gland—DOI: 10.1016/j.jcms.2022.02.007		Excluded	Out of scope
E A Dinces et al. (1997)—Complications of gold weight eyelid implants for treatment of fifth and seventh nerve paralysis.—DOI: 10.1097/00005537-199712000-00008		Excluded	Retrospective design
Jayashankar et al. (2008)—Customized gold weight eyelid implantation in paralytic lagophthalmos—DOI: 10.1017/S002221510800188 [36]	50 patients 33 males, 17 females Average age: 41 years		
J A Lavy et al. (2004)—Gold weight implants in the management of lagophthalmos in facial palsy. DOI: 10.1111/j.1365-2273.2004.00817.x [32]	22 patients (11 males, 11 females) Age range: 23–70 years		
Nunes et al. (2007)—Gold weight implantation: premature and late complications—DOI: 10.1590/S0004-27492007000400008 [35]	20 patients 11 females (55%), 9 males (45%) Age range: 16–86 years (mean 51)		
O’Connell et al. (1991)—Eyelid gold weights in the management of facial palsy—DOI: 10.1017/S0022215100116330 [24]	20 patients 12 females, 8 males Age range: 26–71 years		
T Schrom (2007)—[Lidloading in facial palsy].—DOI: 10.1055/s-2007-966527		Excluded	Comparative study
B Bianchi et al. (2014)—Upper eyelid platinum chain placement for treating paralytic lagophthalmos.—DOI: 10.1016/j.jcms.2014.09.012		Excluded	Retrospective
Carlos Martín-Oviedo et al. (2013)—Hyaluronic acid gel weight: a nonsurgical option for the management of paralytic lagophthalmos.—DOI: 10.1002/lary.23936		Excluded	Retrospective study
Grusha YO, Fedorov AA, Iskusnykh NS, Bogacheva NV, Kobzova MV, Novikov IA, Fettser EI, Shchegoleva TA. [Gold weight implants for lagophthalmos correction in chronic facial nerve paralysis (late results)]. Vestn Oftalmol. 2016 Mar-Apr;132(2):26-32. Russian. doi: 10.17116/oftalma2016132226-32. PMID: 27213794.		Excluded	No English translation available
Biglioli et al. (2020)—Lipofilling of the upper eyelid to treat paralytic lagophthalmos—DOI: 10.1016/j.bjoms.2020.02.017 [45]	75 patients 47 females, 28 males Mean age: 49 years (range 15–80)		
Manodh et al. (2011)—Gold weight implantation as a treatment measure for correction of paralytic lagophthalmos.—DOI: 10.4103/0970-9290.80002		Excluded	Case reports
A Serrat Soto et al. (1998)—[Gold weights for the treatment of lagophthalmos caused by facial paralysis. Our experience and review of the literature]		Excluded	Review article
Izabela Nowak-Gospodarowicz et al. (2022)—The impact of implantation site on procedure success in patients with unresolved facial palsy treated with upper eyelid gold weight loading.—DOI: 10.1038/s41598-022-16169-4		Excluded	Retrospective analysis
Hassan et al. (2005)—Müllerectomy for Upper Eyelid Retraction and Lagophthalmos Due to Facial Nerve Palsy—DOI: 10.1001/archopht.123.9.1221 [47]	34 patients 19 female, 15 male Age range: 10–82 years (average 50)		
S A Kelley et al. (1992)—Gold eyelid weights in patients with facial palsy: a patient review.—DOI: 10.1097/00006534-199203000-00006		Excluded	Review
M El Shazly et al. (2008)—Static management of lagophthalmos following facial nerve paralysis using standardized weights		Excluded	Endpoints not in line with study
Razfar et al. (2009)—Ocular outcomes after gold weight placement and facial nerve resection—DOI: 10.1016/j.otohns.2008.09.028 [38]	22 patients		
Dalkiz et al. (2007)—Gold weight implantation for rehabilitation of the paralyzed eyelid.—DOI: 10.1016/j.ijom.2007.01.023		Excluded	<10 patients
Schrom T et al. (2009)—Patient satisfaction after lid loading in facial palsy—DOI: 10.1007/s00405-009-0981-0		Excluded	
Silver et al. (2009) - Thin-Profile Platinum Eyelid Weighting: A Superior Option in the Paralyzed Eye.—DOI: 10.1097/PRS.0b013e3181a65a56 [37]	100 patients (102 implants) 48 males, 52 females Age range: 8–86 years		
Şahin et al. (2021)—The role of gold-weight implants in the management of paralytic lagophthalmos.—DOI:10.3906/sag-2104-50 [44]	78 patients; 45 males, 33 females Mean age: 51.3 years		
Oh TS, Min K, Song SY, Choi JW, Koh KS (2018) - Upper eyelid platinum weight placement for the treatment of paralytic lagophthalmos: A new plane between the inner septum and the levator aponeurosis. Arch Plast Surg. 2018 May;45(3):222-228. doi: 10.5999/aps.2017.01599. Epub 2018 May 15. PMID: 29788690; PMCID: PMC5968324. [42]	37 patients		
